# Which is the best schedule of autologous blood storage for pre-operative AIS patients? Every week or every 2 weeks

**DOI:** 10.1186/1748-7161-10-S1-O57

**Published:** 2015-01-19

**Authors:** Koji Tamai, Hidetomi Terai, Hiromitsu Toyoda, Hiroyuki Yasuda, Shou Dozono, Yoshikazu Shinohara, Hiroaki Nakamura

**Affiliations:** 1 Dept. of Orthopaedic Surgery, Osaka City University Graduate School of Medicine, Osaka, Japan

## Introduction

It is important to avoid allogeneic blood transfusion for young patients who undergo corrective surgery. Autologous blood transfusion is now the major way to avoid it. We selected a blood storage method among several methods of autologous blood transfusion because there is no need for special equipment. However, progressing pre-operative anemia is still a major concern. We simply compared two different time-schedule of blood storage interval before operation in Adolescent Idiopathic Scoliosis (AIS) patients to know the better way to avoid pre- and post- operative anemia.

## Method

Sixty AIS patients who underwent corrective surgery from 2009 to 2013 in our hospital were included in this study. Inclusion criteria were female, under 20 years old and their first operation. Males, re-operation cases and patients over 20 years old were excluded. All participants successfully stored 1600ml of whole blood before operation. They were randomly divided into 2 groups based on the storage interval. Group 1 consisted of 30 patients who stored 400ml of blood every week and group 2 consisted of 30 patients who stored the same amount of blood every 2 weeks. Blood storage was started 8weeks in group 1 and 4 weeks in group 2 before operation respectively. Blood examination data (RBC, Hb, Hct, etc) were collected and analyzed at pre-operation and 1-, 3-, 7- days after operation. Operation time, estimated blood loss (EBL) and adverse events while storing blood (vomiting, hypovolemic shock, vagovagal reflex etc) were also collected from clinical records prospectively.

## Results

There were no significant differences in age, EBL, RBC, Hb and Hct among two groups before operation. No patients in both groups required allogeneic blood transfusion. Adverse events were 5 in group 1 and 14 in group 2. Changes of the value of Hb were almost same between two groups, however, the value of Hct in group 1 was significantly higher at 3- and 7- days after operation compared to those of group 2.

## Conclusion

Autologous blood transfusion by means of blood storage was effective to avoid allogeneic blood transfusion. As for the blood storage interval, it was proven that every week storage was better than every 2 weeks one in terms of adverse events and recovery of Hct.

